# Attraction, recruitment and distribution of health professionals in rural and remote Australia: early results of the Rural Health Professionals Program

**DOI:** 10.1186/1478-4491-12-15

**Published:** 2014-03-06

**Authors:** Anna L Morell, Sandra Kiem, Melanie A Millsteed, Almerinda Pollice

**Affiliations:** 1 Health Workforce Australia, 400 King William Street, Adelaide, Australia

**Keywords:** Rural and remote health workforce, Nursing, Allied health, Recruitment, Geographic distribution, Australia

## Abstract

**Background:**

Australians living in rural and remote communities experience relatively poor health status in comparison to the wider Australian population (Med J Aust 185:37-38, 2006). This can be attributed in part to issues of access to health services arising from difficulties in recruiting and retaining health professionals in these areas. The Rural Health Professionals Program is an initiative designed to increase the number of allied health and nursing professionals in rural and remote Australia by providing case managed recruitment and retention support services. This paper reports on early analysis of available programme data to build knowledge of factors related to the recruitment and distribution of health professionals in rural and remote Australia.

**Methods:**

Administrative programme data were collected monthly from 349 health professionals over the first 13 months of programme operation. These data were collated and quantitative analysis was conducted using SPSS software.

**Results:**

Sixty-nine percent of recruits were women, and recruits had a mean age of 32.85 (SD = 10.92). Sixty percent of recruits were domestically trained, and the top two professions recruited were nurses (29%) and physiotherapists (21%). Eighty-seven percent were recruited to regional areas, with the remaining 13% recruited to remote areas. Among reasons for interest in the programme, financial support factors were most commonly cited by recruits (51%). Recruitment to a remote location was associated with being domestically trained, having previously lived in a rural or remote location, being a nurse (as opposed to an allied health professional) and older age.

**Discussion:**

The findings provide early support for a case managed recruitment programme to improve distribution of health professionals, and some directions for future marketing and promotion of the programme. It is recommended that an outcome evaluation be conducted to determine the impact of the programme on recruitment and distribution outcomes.

**Conclusion:**

The findings herein begin to address gaps in the literature relating to the effectiveness of interventions to improve the distribution of health professionals. While this provides some preliminary indication that case managed recruitment and retention programmes have capacity to improve distribution, further research and evaluation is required to confirm the impact of the programme on retention.

## Background

### Australia’s health system and the importance of distribution in rural and remote Australia

Australia has a mixed public/private health system which is underpinned by a universal public health funding system called Medicare. According to the World Health Organization [1], the Australian population has a generally good health status in comparison to many other countries, and an average life expectancy at birth of 80 years for males and 84 years for females. However, some groups of the population experience comparatively poor health status, notably those in rural and remote areas and Aboriginal and Torres Strait Islander peoples [2,3].

This can be partially attributed to the fact that Australia is experiencing a shortage of nurses and allied health professionals in rural and remote areas [4], which can limit access to healthcare for communities in these areas. Furthermore, the shortage is likely to be exacerbated in the future due to various factors including an ageing population and growing burden of chronic disease in Australia [5]. The difficulty in recruiting and retaining nursing and allied health professionals in rural and remote areas is a recognised problem [6,7]. Health Workforce Australia (HWA) aims to contribute to addressing this problem through the Rural Health Professionals Program (RHPP).

### The Rural Health Professionals Program

The RHPP was introduced by HWA in January 2012, and is implemented across rural and remote Australia in association with state and territory Rural Workforce Agencies (RWAs)^a,b^. The programme aims to improve the distribution of nursing and allied health professionals from Australia and approved overseas locations by attracting, recruiting and retaining them to work in rural and remote Australia, and in Aboriginal health services. Consistent with the principles of the Commonwealth Code of Practice [8] and the World Health Organization Global Code of Practice on International Recruitment of Health Personnel [9], the programme does not undertake recruitment marketing activities in, or approach health professionals residing in, developing countries.

Various marketing activities are coordinated to attract potential candidates and employers to take part in the programme. Following attraction activities, RWA staff provide a case-managed recruitment service to each recruit by identifying and matching eligible candidates and employers, free of charge for both parties. Recruitment case management involves individually tailored services, such as shortlisting candidates for interview, referee checks, and assistance with obtaining appropriate visas and professional registration. When a candidate is recruited under the programme, they become eligible to receive funded case managed retention support, which aims to support their retention for at least two years. Examples of retention support services that might be provided under the programme include: the provision of psychosocial support; identification and provision of relevant Continuing Professional Development (CPD) opportunities, mentoring or professional supervision; and spousal employment assistance.

The Remoteness Area (RA) structure within the Australian Standard Geographical Classification (ASGC) system (Table 1), as identified by the Australian Bureau of Statistics [10], is used by HWA and RWAs to classify eligible and ineligible positions under the RHPP.

**Table 1:** ASGC-RA classifications

**ASGC - RA classifications**	
RA 1	Major cities
RA 2	Inner regional
RA 3	Outer regional
RA 4	Remote
RA 5	Very remote

Several eligibility criteria apply to both potential programme recruits and the positions to which they can be recruited. Eligibility criteria include:

• The position must be situated in an ASGC-RA 2 to ASGC-RA 5 location, unless a recruit is moving into an Aboriginal health service, in which case the position may be situated in an ASGC-RA 1 to ASGC-RA 5 location.

• If a candidate is already working in a rural or remote location in Australia, they are only eligible for the programme if they move into a position in a more rural or remote location (or into an Aboriginal health service in any location).

• The position must involve at least 50% primary health service delivery.

• The position must be a minimum of 0.4 Full Time Equivalent.

• The candidate must be willing to commit to their position under the programme for a minimum of 12 months.

• The candidate must work in a health profession targeted under the programme (there is a specified list of professions targeted under the programme including, for example, nurses, physiotherapists and social workers).

### Purpose of the paper

According to Dolea et al. [11], there is a significant gap in the literature in relation to the effectiveness of interventions designed to improve the distribution of health professionals. Specifically, almost all of the interventions that have been evaluated are targeted towards medical professionals. Interventions to improve the distribution of nursing and allied health professionals remain largely unstudied. In addition, the majority of evaluated interventions designed to support recruitment and retention of health professionals have been education programmes (for example, programmes that require clinical placement rotations in rural or remote settings), rather than financial incentive or professional and personal support programmes.

The purpose of this paper is to begin to address these gaps by detailing the early results of the RHPP. Given the early stage of the programme and the fact that it aims to retain recruits rurally/remotely for at least two years, it is premature to report on retention outcomes. However, this paper does examine and report on the recruitment and distribution of health professionals under the programme to date. In exploring this, the paper seeks to answer the following four key research questions:

• Who was attracted and recruited to work in rural and remote Australia as part of the RHPP?

• Why were recruits interested in participating in the RHPP?

• How has the RHPP influenced distribution of nursing and allied health professionals?

• What factors influence the distribution of nursing and allied health professionals under the RHPP?

## Methods

Administrative programme data detailing demographic and professional information of the candidates recruited to the programme were routinely collected on a monthly basis by RWAs implementing the programme. In total, data were collected from all 349 programme recruits over the period 1 January 2012 to 28 February 2013. The re-identifiable data were collated by HWA and quantitative data analysis was undertaken using the Statistical Package for the Social Sciences (SPSS) software. Independent samples t-tests were used to examine the relationships between age of recruits and other variables, and chi-square tests were used to explore relationships between a range of other variables.

## Results

### Who was attracted and recruited to work in rural and remote Australia as part of the RHPP?

Over the period 1 January 2012 to 28 February 2013, a total of 349 nursing and allied health professionals were recruited under the RHPP. Sixty-nine percent (N = 242) of recruits were women and 31% (N = 107) were men. The average age of candidates was 32.85 years (SD = 10.92) and more than half (65%, N = 227) of all recruits were aged less than 34 years at the time of their recruitment.

The number of those recruited who obtained their primary health-related qualification in Australia was 210 (60%), and 138 (40%) obtained their primary health-related qualification overseas.

Table 2 lists the number and proportion of recruits across each of the eligible professions for the programme.

**Table 2:** Number and proportion of recruits across eligible professions

**Profession**	**Recruits (N)**
Nurse	100 (29%)
Physiotherapist	75 (21%)
Dentist	37 (11%)
Social worker	30 (9%)
Occupational therapist	24 (7%)
Pharmacist	23 (7%)
Psychologist	13 (4%)
Dietitian	10 (3%)
Podiatrist	6 (2%)
Other^a^	31 (9%)
Total	349^b^

^a^‘Other’ includes Aboriginal health workers, audiologists, chiropractors, exercise physiologists, mental health professionals, midwives, optometrists, osteopaths, radiographers and speech pathologists.

^b^Percentages add up to 102% due to rounding.

Forty-nine percent (N = 172) of recruits indicated that they have spouses and 35% (N = 121) said they had at least one child. Forty-six percent (N = 162) said they had never previously lived in a rural or remote area prior to joining the RHPP, while 24% (N = 83) were living in a rural or remote area at the time they joined the programme. Thirty percent (N = 104) of all recruits were new to the workforce and had not previously been employed as a health professional.

### Why were recruits interested in participating in the RHPP?

On joining the RHPP, recruits were asked to specify why they were interested in the programme. Their responses were categorised for further analysis as follows:

1. Financial factors (for example, financial support for relocation and financial incentives).

2. Professional factors (for example, job opportunities, accreditation and registration support, professional support and the ability to access CPD).

3. Location factors (for example, rural lifestyle and community).

4. Family factors (for example, family support and opportunities for family).

5. Other factors or unknown (also includes a small number who specified support for obtaining a visa and support for orientation into the new workplace or community).

Table 3 shows the number and proportion of recruits that referred to each of these categories in describing why they wanted to join the programme.

**Table 3:** Recruits’ reasons for interest in participating in the RHPP

**Reasons for interest**	**Recruits (N)**
Financial factors	177 (51%)
Professional factors	125 (36%)
Location factors	127 (36%)
Family factors	32 (9%)
Other factors or unknown	56 (16%)

Chi-square analyses were conducted to determine whether there were any statistical relationships between reasons for interest in the RHPP and gender, internationally/domestically qualified, profession (nursing or allied health), rural background and whether the candidate has a spouse or children. A significant relationship was found between whether the recruit had previously lived in rural or remote Australia and whether they cited location factors as a reason for interest, such that those who had previously lived in these areas were less likely to mention location factors (29% cited location factors) and those who had not previously lived in these areas were more likely to mention location factors (45% cited location factors) as a reason for interest in the programme (Χ^2^(1, *N* = 349) = 9.82, *P* <0.01). In addition, nursing professionals were significantly more likely than allied health professionals (16% of nurses as opposed to 7% of allied health professionals) to mention family factors as a reason for interest in the programme (Χ^2^(1, *N* = 329) = 7.34, *P* <0.01). No statistically significant relationships were found between reasons for interest in the programme and: recruits’ gender; whether a recruit was classified as internationally or domestically trained; whether they had a spouse; or whether they had children.

Independent samples t-tests were conducted to determine whether there were relationships between reasons for interest in the RHPP and age of recruits. Recruits who cited family reasons were, on average, significantly older (*M* = 38.41, *SD* = 7.87) than those who did not mention family reasons (*M* = 32.29, *SD* = 11.03; *t*(44.42) = -4.02, *P* <0.01; 95% CI (-9.18, -3.05)). The magnitude of the difference between means, however, was quite small (η^2^ = 0.04). No other significant age differences were found.

### How has the RHPP influenced distribution of nursing and allied health professionals?

As shown in Table 4, the majority of RHPP recruits (87%, N = 304) were recruited to inner-regional and outer-regional areas (ASGC-RAs 2 and 3) along with the proportion of the Australian population residing in these areas for context [12].

**Table 4:** Distribution of RHPP recruits

**ASGC-RA recruited to**	**Recruits (N)**	**Proportion of Australian resident population**
ASGC-RA2	150 (43%)	19%
ASGC-RA3	154 (44%)	9%
ASGC-RA4	30 (9%)	1%
ASGC-RA5	15 (4%)	1%
Total	349	30%

Table 5 shows the locations of recruits’ previous employment, and where they were distributed to as a result of the RHPP.

**Table 5:** Locations of recruits’ previous employment and distribution as per RHPP placement

	**ASGC-RA location of position under RHPP**				
**Location of previous employment**	**ASGC-RA2**	**AGSC-RA3**	**AGSC-RA4**	**AGSC-RA5**	**Total**
New to the workforce	47 (45%)	44 (42%)	12 (11%)	2 (2%)	105 (100%)
Overseas	46 (43%)	57 (53%)	3 (3%)	1 (1%)	107 (100%)
ASGC-RA 1	51 (51%)	36 (36%)	8 (8%)	5 (5%)	100 (100%)
ASGC-RA2 or RA3	5 (15%)	16 (48%)	6 (18%)	6 (18%)	33 (100%)
ASGC-RA4 or RA5	1 (25%)	1 (25%)	1 (25%)	1 (25%)	4 (100%)
Total	150 (43%)	154 (44%)	30 (9%)	15 (4%)	349 (100%)

### What factors influence the distribution of nursing and allied health professionals under the RHPP?

Chi-square analyses were used to examine what factors are related to RHPP recruits taking up a rural (ASGC-RA2 and ASGC-RA3) or remote position (ASGC-RA4 and AGSC RA-5). Recruits that had previously lived rurally or remotely were more likely to take up a remote position (18% of those that had previously lived rurally or remotely took up a remote position) and those who had not previously lived in these areas were less likely to take up a remote position (only 7% who had not lived rurally or remotely took up a remote position; Χ^2^(1, *N* = 349) = 8.10, *P* <0.01). Nurses were also more likely to take up remote positions (20% of nurses took up remote positions) compared with allied health professionals (only 10% of allied health professionals took up remote positions; Χ^2^(1, *N* = 329) = 6.52, *P* = 0.01). Internationally trained recruits were less likely to take up remote positions (7% of internationally trained recruits took up remote positions) when compared to domestically trained recruits (17%) (Χ^2^(1, *N* = 349) = 8.25, *P* <0.01). In terms of candidate age, participants who took up remote positions tended to be older (*M* = 36.69, *SD* = 13.35) than those who took up rural positions under the RHPP (*M* = 32.28, *SD* = 10.42; *t*(52.26) -2.12, *P* <0.05; 95% CI (-8.58, 0.24)). The magnitude of the difference between means was very small (η^2^ = 0.01). Statistically significant relationships were not found between recruits’ choice of a rural versus remote position and: gender; whether they had a spouse; whether they had children; and their reasons for interest in the programme.

## Discussion

This research provides a profile of those who were attracted and recruited to work in rural and remote locations throughout Australia as part of a case managed, government-funded recruitment and retention programme, and an early indication of the factors that impact on their distribution.

These early findings may also have some implications for the future focus and promotional activities of the programme. For example, this research suggests internationally trained recruits may be less attracted to work in remote areas compared with domestically trained recruits, and older (likely more experienced) recruits may be more attracted to work in remote areas.

Previous research [13] has shown that health professionals trained rurally and/or remotely are more likely to be recruited and retained in rural and remote areas. Indeed, 54% of RHPP recruits said that they had previously lived in a rural or remote area prior to joining the programme. However, the research has also to some extent, demonstrated the capacity of a case-managed recruitment and retention programme to have an impact on the distribution of allied health and nursing professionals throughout rural and remote Australia. Forty-six percent of RHPP recruits had never lived in rural or remote Australia and 100 health professionals moved from practising in metropolitan Australia to practising in rural and remote locations with the support of the RHPP. Overall, after only 13 months of programme operation, 15 recruits had also commenced practice in very remote locations, despite only four recruits reported as having worked in remote or very remote locations prior to joining the RHPP. Nevertheless, it is imperative going forward that an evaluation be conducted that compares the number of health professionals that are recruited to rural and remote Australia with, as well as without, the support of the programme. Such comparisons are not possible using administrative programme data alone, but would enable rigorous identification of the capacity of case-managed recruitment programmes to impact on recruitment and distribution outcomes.

In addition, given the early stage of the programme, it is not yet possible to determine the extent to which it has impacted on retention outcomes. In the future, this would ideally be measured through the identification of an appropriate control group that allows for comparison of retention outcomes for people who are employed and case-managed under the RHPP with those that are employed in rural and remote locations but are not part of the RHPP. Future research and evaluation could also consider whether there are specific aspects or support services offered as part of the programme that contribute more strongly to successful recruitment and/or retention of health professionals.

## Conclusions

This paper has begun to address some of the significant gaps in the literature regarding the effectiveness of interventions specifically designed to improve recruitment and distribution of allied health and nursing professionals. The early findings presented here suggest that case managed recruitment and retention programmes can attract these professionals to work in both rural and remote locations and offer some preliminary directions for promotional targeting of the programme going forward. Nevertheless, it is imperative that further research and evaluation identify programme recruitment, distribution and retention outcomes in order to inform evidence-based programme and policy recommendations into the future.

## Endnotes

^a^RWAs are not-for-profit organisations funded by the federal government as well as their respective state/territory governments to provide recruitment services, support services and workforce planning in rural and remote communities.

^b^The Australian Capital Territory is classified as ASGC-RA 1 and therefore is not eligible for participation in the RHPP.

## Abbreviations

ASGC: Australian Standard Geographical Classification; CPD: continuing professional development; HWA: Health Workforce Australia; RA: remoteness area; RHPP: Rural Health Professionals Program; RWA: Rural Workforce Agency; SPSS: Statistical Package for the Social Sciences.

## Competing interests

The authors are employed by Health Workforce Australia, the organisation which funds and manages the Rural Health Professionals Program, and which is financing the manuscript.

HWA is an Australian Government initiative; its role is to provide a national, coordinated approach to health workforce reform. It was established by the Council of Australian Governments to address the challenges of providing a skilled, flexible and innovative health workforce that meets the needs of the Australian community.

## Authors’ contributions

ALM and SK manage the Rural Health Professionals Program, and collected the data. ALM, SK, MAM and AP drafted the manuscript. MAM carried out the statistical analysis. All authors read and approved the final manuscript.
